# Successes and challenges of China’s health care reform: a four-decade perspective spanning 1985—2023

**DOI:** 10.1186/s12962-023-00461-9

**Published:** 2023-08-30

**Authors:** Mihajlo Jakovljevic, Hanyu Chang, Jay Pan, Chao Guo, Jin Hui, Hao Hu, Danko Grujic, Zhong Li, Lizheng Shi

**Affiliations:** 1 Institute of Comparative Economic Studies, Hosei University Faculty of Economics, Tokyo, Japan; 2 Department of Global Health Economics and Policy, University of Kragujevac, 34000 Kragujevac, Serbia; 3 Institute of Advanced Manufacturing Technologies, Peter the Great St. Petersburg Polytechnic University, Saint Petersburg, Russian Federation; 4 Department of Medical Insurance, The First Hospital of Lanzhou University, Lanzhou, China; 5 HEOA Group, West China School of Public Health and West China Fourth Hospital, Sichuan University, Chengdu, China; 6 School of Public Administration, Sichuan University, Chengdu, China; 7 Institute of Population Research, Peking University, Beijing, China; 8 School of Economics and Management, Zhejiang Sci-Tech University, Hangzhou, China; 9 Institute of Chinese Medical Sciences, University of Macau, Macao, SAR China; 10 Clinic for Cardiac Surgery, University Clinical Center of Serbia, Belgrade, Serbia; 11 School of Health Policy and Management, Nanjing Medical University, Nanjing, Jiangsu China; 12 Health Systems Analytics Research Center, Tulane University, New Orleans, USA

**Keywords:** Chinese health reform, Standards, Perspective, Market, Supply, Demand, Medical care, Pharmaceuticals, Medical devices, Industry, Manufacturing, Insurance policy

## Abstract

Chinese health system remains the crucial one for understanding the wider healthcare landscape across the Global South and in particular the leading Emerging Markets. Purpose of our observation was to understand the inner dynamics of mainland Chinese health reforms adopting a lengthy time horizon. We have analysed the public reports and seminal evidence on Chinese of multiple waves of national health reforms taking place since 1980s in terms of medical care and pharmaceuticals provision and financing. Chinese international trade with ASEAN nations and wider South-East Asia is accelerating its growth after the recovery of trade routes. In terms of health sector this means that global demand and supply of medical goods, services and pharmaceuticals remains largely driven by Chinese domestic developments. Furthermore, Chinese domestic manufacturing and sales of decent quality medical devices and services have grown exponentially. Some temporary pitfalls and increasing in rural–urban inequalities in equity of access and affordability of medical care and pharmaceuticals did take place. Despite these difficulties to generate a balanced development strategy for the largest global market, this is a clear path upwards. Further upcoming improvements expanding health insurance coverage are in strong demand for certain layers of the society. Domestic bottleneck weaknesses yet remain manufacturing, import and market penetration of cutting-edge pharmaceuticals such as monoclonal antibodies and targeted oncology agents. Yet some of these obstacles are likely to be overcome in foreseeable future with the adoption of responsible strategies by governmental agencies in health care arena.

## Chinese health sector in the wider BRICS emerging markets context

According to leading Brazil, Russian, India, China, South Africa (BRICS) countries reports and multilateral agencies and Brookings Institute releases, these Emerging Markets remain the engine of real gross domestic product growth [1, 2]. Their GDP growth rates and unemployment rates showed steadily higher resilience compared to G7 throughout the previous global economic crisis 2008–2016 [3, 4]. The similar patterns of strong resilience against halted global economic growth were exposed during the Pandemics triggered crisis in trade supply routes worldwide [5]. This self-reliance on domestic market demand and supply was particularly prominent in China as the wealthiest economy of the group. Home-born care for the elderly in an era of shrinking family caregiving due to aging of populations is another challenge of fiscal sustainability to be faced by all BRICS. All five members expose vulnerabilities in medical care delivery to their vast rural and remote communities far away from urban and industrialized coastal and megalopolis areas. Additional bottleneck weakness is increasing socioeconomic inequalities between the rich and the poor. This is witnessed by Gini indexes and great out-of-pocket spending in all of the BRICS [6, 7]. Yet these and many other allocation inefficiencies are being gradually overcame by complex adaptive strategies. It is worthy to note that four of these nations with the partial exception of India [8], managed to continuously increase their total health expenditure expressed as share of GDP spending since 1995 to contemporary momentum [9]. Thus, there are reasonable grounds for optimism in terms of improved access to health care and essential medicines in these crucial LMICs nations. The relevance of BRICS nations for the global health care arena is well documented with recent forecasts of their future expenditure patterns until 2025and 2030 [10, 11]. We shall study the Chinese health reform pathway in greater extent for the fact it has by far the largest global impact to medical supply and demand. [12]

## Contemporary reform of Chinese health care system since 1980s

Reform of Chinese health care system began in 1985, when the former Chinese Ministry of Health’s Report on Several Policies Concerning Health Reform proposed the principle of “relaxing policies, streamlining administration and delegating powers, raising funds from various sources, broadening the horizon in developing health undertakings and doing a good job” [13], which represents the official beginning of China’s medical system reform. The medical funds, during this period, are covered by the state and enterprises. Lack of access to financing and prevention measures in all sides results in issues such as hospitals generating rapid growth of medical expenses and unreasonable allocation of medical resources [14]. What’s more, the survey suggested that the average medical expenses of employees in state-owned enterprises increased from 35.46 yuan in 1978 to 63.61 yuan in 1985, with an average growth rate of 8.7 percent [15]. In 1994, the Chinese government launched a pilot program of social medical insurance that combines social guarantee with individual payment in Zhenjiang, Jiangsu province and Jiujiang in Jiangxi Province. And 1998 witnessed the launch of the reform of urban employees' medical insurance, which marked that China's social medical security system starts to adapting to the socialist market economy. In October 2002, the “Decision of the CPC Central Committee and The State Council on Further Strengthening Rural Health Work” clearly pointed out that a new rural cooperative medical system, which focuses on planning serious diseases costs as a whole, should be gradually in place. In 2003, the outbreak of SARS exposed the drawbacks of China's emphasis on clinical practice over public health. Since then, the government began to realize the importance of a sound health system for social and economic development and thus expedited the reform of China's health system [16]. In 2005, the model of separating management from operation of hospital was in place, mainly represented by Shanghai Shenkang Hospital Development Center and Jiangsu Wuxi Hospital Management Center. At that time, the Development Research Center of The State Council issued a research report on medical reform, that China's medical and health system reform is unsuccessful. That represents a turning point for China's medical system reform. From 2006 to 2008, the Chinese government gathered various forces to explore the reform of the medical and health system and solicited opinions from the public. In 2009, China embarked on reform of the new medical and health care system that aimed at making medical care affordable and accessible and delivering access to health care and financial protection. The Chinese government has proposed that by 2011 the basic medical security system will cover all urban and rural residents, a national system of essential drugs will be initially established, the community-level medical service system will be refined, equal basic public health services will be in place, and public hospitals will be reformed on a trial basis. In 2010, China identified 16 pilot cities and set 37 provincial-level pilot regions to carry out the pilot reform of public hospitals. In 2011, the drug sales of zero margin were fully implemented in community-level medical and health institutions run by the government, and a basic national drug-supply system was initially established. In 2012, the comprehensive reform of county-level public hospitals was started, which is a key link in delivering an accessible and affordable medical treatment in rural areas, and will be fully in place in 2015.

In 2016, China integrated the medical insurance for non-working urban residents and the new rural cooperative medical insurance into a unified medical insurance system for both non-working urban and rural people. In 2017, the comprehensive reform pilot of urban public hospitals was fully carried out, with 93.9 percent of urban public hospitals canceling drug markup, effectively controlling the unreasonable growth of medical expenses in public hospitals. In May 2018, the National Healthcare Security Administration was formally established as an institution directly under The State Council. China’s medical security undertakings entered the stage of development in a more standardized way, with comprehensive reform of public hospitals, canceled drug markups, official drug purchase with volume, DRGS and other new policies. By the end of 2020, China had 1.36 billion people participating in full-coverage basic medical insurance, with its coverage over 95 percent [17]. And a basic health care system covering both urban and rural residents will be in place. We have established comparatively sound public health service system, medical service system, medical security system, standard drug supply security system, scientific management and operational system of medical and health institutions, formed a pattern of diversified medical system, ensured that everyone have access to basic medical and health services, and their multi-level medical and health care needs are satisfied. The Chinese people enjoy a better health condition. Since then, reform of Chinese health care system enjoyed steady development.

In the course of the new reform of medical and health care system, Sanming, a city of Fujian province, as one of the first batch of pilot cities for graded diagnosis and treatment and the construction of medical consortium, has promoted the reform of medical insurance payment methods by forming a close medical consortium as the carrier, and has made important explorations for the key work and pilot work plan of the construction of the graded diagnosis and treatment system. Thus, a model of systemic reforms called the Sanming model has been pointed out as a quintessence for deepening medical reform because of its success to address the inefficiency and waste at public hospitals [18, 19]. The success of the Sanming’s health care reform is inseparable from the support of the central government, while also providing lessons for other places to learn [20]. The Sanming’s experience was even praised by President Jinping Xi at a key meeting of the central leading group for deepening overall reform in 2017, urging promotion of its practices nationwide. The National Health Commission has issued a notice summarizing the experience of graded diagnosis and treatment and medical consortium construction in Sanming City and requiring all provinces of the country to promote the Sanming model.

**Fig. 1:**
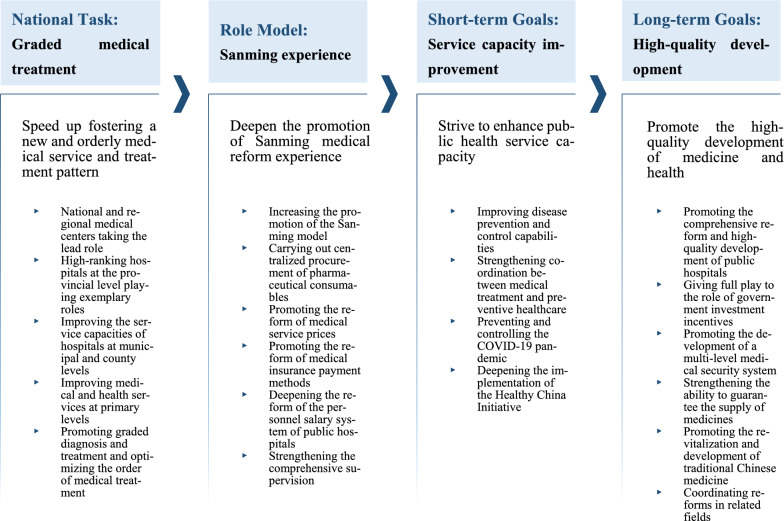
A framework of the key tasks of deepening overall reform of the medical and health care system in 2022

In 2020, further promoting the experience of medical reform in Fujian Province and Sanming City has been included in the key tasks of deepening the reform of the medical and health system listed by a notice released by the General Office of the State Council each year since 2012. In 2021 and 2022, “further promoting the experience of medical reform in Sanming City” has even been regarded as one of the four key tasks of the notice for two consecutive years. By clarifying the overall requirements, key tasks and work arrangements, the notice draws a roadmap for further reform (Fig. 1), to promote the shift from treatment-centered to people’s health-centered, and accelerate the Joint Reformation for Public Health Services, Medical Insurance, and Medical Production-Circulation.

As regards Chinese medical system, before 2009, the main issue is whether liberalize it or not. Some believe that the root cause of expensive and difficult medical care is the over-marketization resulted from the lack of investment in hospitals by the government [21], while some others contend that it is because of severely insufficient supply of medical care service and security [22]. Since 2009, the government has tripled its spending on health care, with the focus on solving the problem of high cost and difficulty in getting medical care.

Compared with other countries, China has a larger population, more complex health system as well as its reform, and there is more distinctive features between regions. In the past decade and more, China's health system reform has made great progress, but there are still many challenges. First, China has made significant progress in making health care more equally available and strengthening financial protection, especially for low-income people. Second, there still remain many things to be done in non-communicable disease control, improving delivery efficiency, health expenditure control, and we need to make more efforts in mitigating uneven distribution of health resources, meeting increased demand for quality and payed service, and improving public [23]. Given China's commitment to achieving universal health and Building Healthy China in 2030, the country needs to continue the process and take necessary policy measures to ensure it works. [24]

## Main morass of Chinese health care system in recent years

First, with the urbanization process, the medical security needs of rural and floating population are increasingly expanding, which cannot be well met. Urbanization affects the allocation of medical security resources, especially the inflow of rural population into cities, which includes two challenges. On the one hand, in rural areas, the elderly, women and children have a large scale. How to better meet their needs for medical security services is a major challenge for the supply of rural medical security services. On the other hand, the scale of floating population closely related to urbanization is large. In 2019, the number of people separated from households across the country reached 280 million, including 236 million floating population [25]. The settlement of off-site medical treatment is related to the medical treatment security rights and interests of the groups in cross-medical insurance areas including the floating population. In particular, the process of off-site medical treatment settlement of outpatient expenses needs to be further accelerated [26]. If we continue to strengthen the direct settlement of medical treatment in different places, will it solidify the current level of medical insurance overall planning, which is not conducive to the construction of medical insurance integration? This is also an important issue to be considered in the future medical security reform and optimization.

Second, the contradiction between the gradual expansion of the elderly population and the sustainable development of basic medical insurance funds. It is predicted that, China’s population aged 60 and above will rise from 263 to 522 million from 2020 to 2050, especially after 2030, the proportion of the elderly aged 80 and above will increase rapidly, which is expected to reach 54.48 million in 2030 and 133 million in 2050. As a result shows, about 47–63 years old is the age range with the fastest growth in the total medical expenses, and there will be a rapid increase in the total medical expenses from the age of 71 to the time of death, which will bring great pressure on the expenditure of the basic medical insurance fund [27]. Moreover, the basic medical insurance payment responsibility structure of the elderly group is difficult to adapt to the needs of the continuous large-scale elderly group. On the one hand, the elderly group of urban workers who have paid for 15 consecutive years before reaching the statutory retirement age need not bear the responsibility for payment after retirement. On the other hand, the proportion of individual payment responsibility of the elderly groups of urban and rural residents is low, and they rely heavily on government finance.

Third, uneven distribution of public health resources among regions. Although the Chinese government inclines the public health expenditure to the poor provinces in central and western regions, the imbalance and inequity in the allocation of public health resources among regions are still expanding. China has carried out a comprehensive reform to alleviate the increasingly obvious inequities in public health and medical services among regions, which has made the public health expenditure increase significantly in poor provinces from the central and western regions. The per capita public health expenditure has increased in all 31 provinces of China since 2008. 28 Among these provinces, Hainan, Jiangxi, Guizhou and Guangxi are the top four provinces in terms of per capita public health expenditure growth, with a growth rate more than 400%, all of which are relatively poor provinces [28]. In addition, the proportion of provincial public health expenditure in provincial GDP and local fiscal expenditure has also increased significantly in central and western provinces. Obviously, the Chinese central government has invested a large amount of funds into the central and western provinces by transfer payment, which causes a large growth in public health expenditure in these provinces [28]. However, it is still difficult to alleviate the imbalance and inequality of public health resources allocation only by increasing the public health expenditure in central and western regions. The resource of medical and health institutions and health personnel shows a decreasing trend from east to west both in 2008 and 2018 [28]. In 2008, Beijing, Shanghai and Tianjin are the top three provinces with the most abundant public health resources, while Tibet, Xinjiang and Inner Mongolia are the bottom three ones. And the ranking of public health resource is still similar in 2018. Moreover, the imbalance and inequity in the allocation of public health resources among regions are still expanding. For example, the quantity of health personnel per thousand square kilometers per million people in Beijing has increased from 517.83 in 2008 to 724.40 in 2018, while that in Tibet has increased from 2.62 to 4.51 in the same period. It means that the gap of public health resources between Beijing (with the most abundant public health resources and Tibet (with the least public health resources) is still expanding during 2008–2018.

Forth, there are a lot of waste of resources in public health system and medical insurance system which seriously reduces the efficiency of public health expenditure in China. Chinese government has carried out the market-oriented reform of governmental hospital, and requires these hospitals to be responsible for their own profits and losses. Thus, the responsibility for making profits is mainly shared by all doctors, which will lead to doctors treating minor diseases in the way of treating serious diseases, requiring patients to participate in various physical tests, prescribing a large number of safe, inefficient and high profit drugs, and finally making the patients and the medical insurance system pay the bill. At the same time, many governmental hospitals increase their income through the economies of scale, for example, the number of beds in the First Affiliated Hospital of Zhengzhou University is more than 10,000. Such a large-scale hospital is almost unimaginable in the United Kingdom and the United States, but there are many in China. Although it brings convenience for residents to get medical services, it will not only cause a great waste of resources, but also could not bring residents a higher sense of gain, because such a large hospital needs residents and medical insurance system to bear most of the costs. The ultimate result is a great waste of resources, medical insurance fund deficit, low sense of patient acquisition, which all reflect the low efficiency of Chinese public health expenditure.

## Strategies to overcome obstacles in provision of equality in access to medical care in rural and urban provinces

China’s social economy has made great progress since the reform and opening up, but there has always been disparities between urban and rural development, particularly in basic medical services, which is mainly caused by disparities in government investment, medical security system, regional economic growth and residents' income, which are mainly reflected by the disparity in health care financing, distribution of medical resources, medical security level, health consumption concept, and the impact on people’s health as well as the overall development of the society [29].

What is equalization of medical and health services? Chinese scholars are divided in the understanding of it. Fenghua Yu holds that it means people could enjoy equal access to a healthy and quality life [30]. Zhiqiang Wang believes that it is necessary to give the masses access to the most basic and roughly the same medical and health services despite the differences [31]. Jing Xia contends that medical and health equalization is a relative concept, which does not mean the exact same, but the goal of maximizing the right to health, to achieve the combination of fairness and efficiency of medical and health services with distribution according to needs so that we can deliver desired medical service to our people. [32]

Given China's large population, disparities in regional growth and the development gap between urban and rural areas, there is inequality in medical and health services among people, between regions and between urban and rural areas. Jinwei Liu’s survey shows that there is a sharp contrast between urban and rural areas in Beijing due to huge difference in income of urban and rural residents, the type of medical insurance and the accessibility of medical resources among other factors [33]. And there is an obvious disparity between urban and rural residents in the utilization of paid medical and health services. Biao Xie’s survey of Wuhan city indicates that there are significant differences between urban and rural areas in the quantity and quality of medical institutions, as well as the quantity and technical skills of medical practitioners [34]. Xiong Yue’s research, based on CHARLS data in 2013, suggested that, after excluding reasonable differences, there was serious inequality of opportunity in the utilization of medical services in urban and rural areas, mainly affected by the varied guarantee level given by the government [35]. A survey conducted by Wenli et al. In Ningxia Autonomous Region to study the northwest China showed that there was a huge gap between urban and rural areas in terms of medical care expenditure in total, medical care expenditure per capita, medical resource distribution and residents' health status, which was mainly affected by resource allocation pattern and supervision system [36, 37]. The investigation conducted by Guo et al. shows that there are significant differences between urban and rural areas in medical insurance coverage, funding, resource allocation, and policy operation effect, and the main contributing factors are financial burden, non-communicable diseases, and the number of population over 65 [38].

In China, a developing country with rapidly growing social economy, equal access to health care is highly concerned by government departments and researchers. In 2008, the Chinese government proposed the strategic goal of “delivering everyone access to basic medical and health services”, and made a full-plan to establish a basic medical and health system and provide the Chinese people a healthier life. Since 2010, the Chinese government started providing equitable primary health care services to the its people with the aim of delivering a fairer health service. The new health system reform carried out in China in 2009 is also designated to deliver affordable and accessible medical care to the public, to ensure that everyone enjoys a fair and basic medical care services. On top of the reform, the Chinese government implemented universal health insurance system, hierarchical diagnosis and treatment system, reform of public hospitals, family doctors agreement, and the basic drug system. Take the medical insurance system as an example, China is successful in providing financial support to people with varied ability to pay for medical care. For employees, the insurance premium is jointly paid by the employer and the individual is 12 to 9, while for non-working urban residents and rural residents, the insurance cost is jointly paid by themselves and the government. In this way, the full coverage of medical insurance is ensured.

In recent years, China’s urban and rural medical and health systems have been improved, the gap between urban and rural areas has been narrowed, and the reform of basic medical and public health systems has been deepened. The Chinese government has built a more effective long-term system that prevents poverty caused by illness by ensuring that there are doctors, hospitals, and institutional guarantee for rural poor patients and deliver a healthier life to them all. China built a system that covered all poverty-stricken people under the triple system of basic medical insurance, serious disease insurance, and medical assistance. Above all, China gradually overcome obstacles in provision of equality in access to medical care in rural and urban provinces and make a great success.

## Competition and private hospitals development in the hospital market

Decades ago, the trend of market-oriented reform in the healthcare industry swept dramatically over the world, especially in developed regions, such as Europe and North America [39, 40].This phenomenon was driven by a series of factors, including growing health demand, the increasing need for high-quality medical services, and the pressure of inflation in healthcare expenditure. The specific measures for the market-oriented reform mainly focus on expanding the patients’ autonomy and increasing the suppliers [41]. The former is the way mainly used by European countries, such as the United Kingdom, while China currently adopts the latter.

In 2009, a new round of comprehensive and nationwide healthcare reform was launched by the Chinese government with the aim to solve the problems summarized as getting medical is difficult and expensive (*kan bing nan, kan bing gui*) [42]. With this reform, a range of market-oriented policies was introduced into the healthcare industry to enhance the availability of healthcare to meet the people’s diversified needs and intensify competition among hospitals to improve their quality and efficiency [42]. The mainly specific policies include as follows: (1) separating the operational system within public hospitals administration from the surveillance of governmental regulatory division [43]; (2) relaxing the entrance of private investments in the healthcare system [44]; (3) switching governmental role in the financial model of the healthcare system from supplementary suppliers to supplementary demanders, namely medical insurance. [45]

**Fig. 2:**
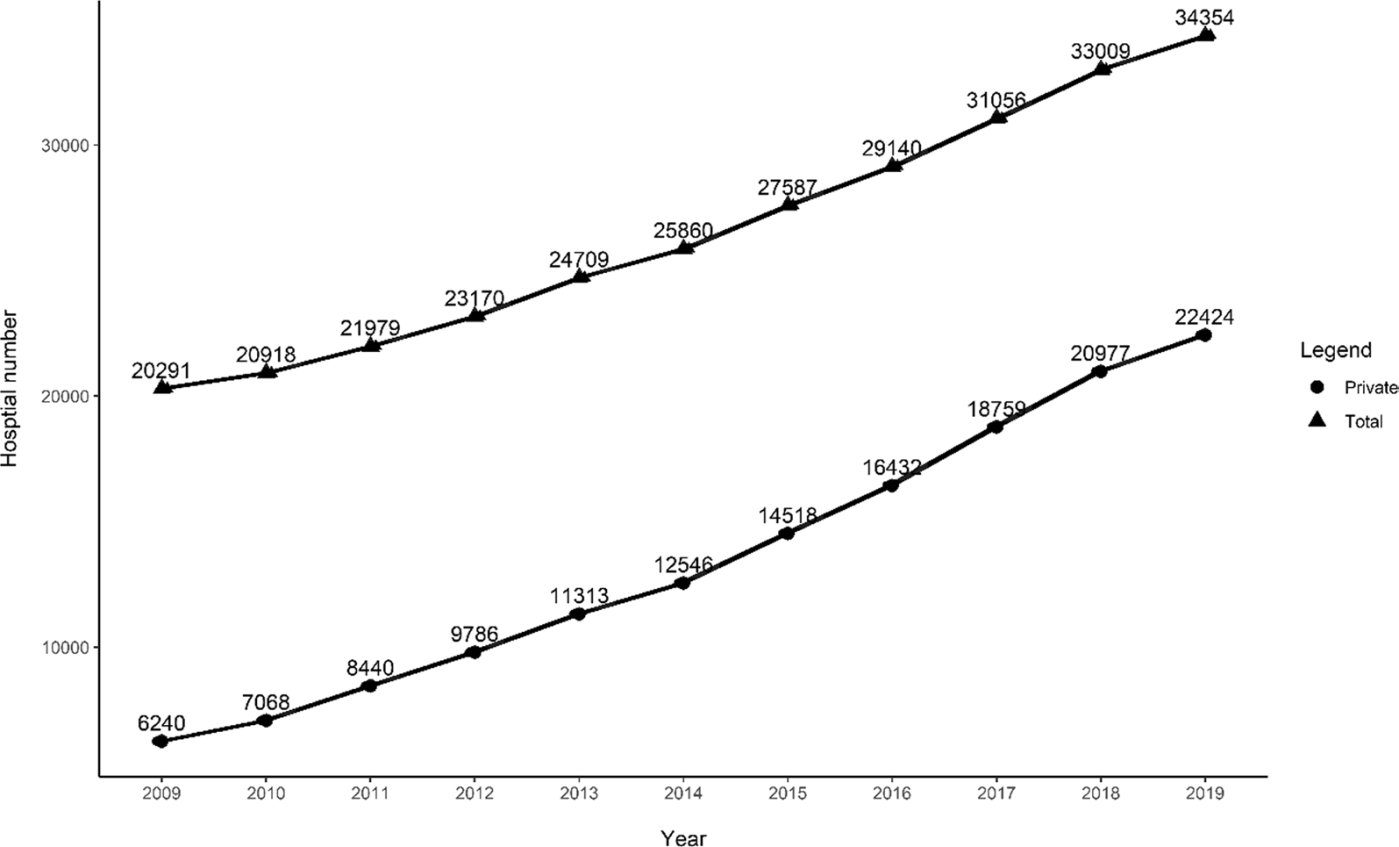
Hospital number, China, 2009 to 2019

With the implementation of pro-competition policies, the hospital market, a most important market in the healthcare industry and also the main focus of this market-oriented reform, changes dramatically no matter in scale and the constitutes [46]. Use the number of hospitals to briefly describe the changes in the hospital market, as shown in Fig. 2 below, the total number of hospitals increased from 20,291 in 2009 to 34,354 in 2019; the private hospitals expanded rapidly and their numbers exceed the public ones in 2015.

The debate, however, for the role of competition and private hospitals in the healthcare system is still heated and gradually evolved into two factions: pro-market and pro-government groups [47]. The former believe that better internal management and lower operational cost would facilitate higher efficiency of private hospitals, and the competitive pressure brought by the increase in private hospitals would push public ones to painstakingly take measures to attract patients, such as improving the management level and efficiency, which in turn to drive the decrease in medical costs and improve the service quality [48–50]. For the advocates of pro-government, they think the obvious differences between the healthcare market and the general goods market in the textbooks would result in market failure, and even impair the patient’s welfare [51, 52]. For example, the serious information asymmetry between physicians and patients provides the opportunity for the hospitals or the physicians to take “unwanted behavior” in the particular system context (such as the unreasonable design in financial incentives), including induced demand, over-prescribe [53]. When facing competition pressure, the hospital could act differently from general goods suppliers, and the phenomenon of “medical arm races” was the main negative effect of the hospital competition criticized by many scholars in pro-government groups [54].

Due to the particularity and complexity of healthcare, it is not possible to obtain obvious or persuasive evidence simply from theoretical inference. Empirical evidence based on the retrospective data in the real world was provided by many studies. Most evidence, especially for empirical evidence, was produced based on the developed countries or regions [55–59]. Due to the distinct between China and developed countries, such as the design of the healthcare system, and economic level, evidence based on developed countries cannot be directly used for the analysis of China’s healthcare industry. Even for the U.S., in fact, which carries out many related studies, the conclusion for the role of market-orient reform is still inconclusive, and the debate still exists. Due to the imperfection of the medical information system, the data for health services is limited, hindering the related research in China. To our knowledge, most evidence based on China discussed the market-oriented reform or the role of competition as well as private hospitals from the theoretical perspective, and empirical evidence just based on the Yearbook regional data. Although several studies were conducted at the patient individual level, they are still insufficient to solve the above debate and cannot provide enough implications for the authorities.

In recent years, the focus of debate has gradually shifted from the effectiveness of the competition and private hospitals to the role of the government or the authority in the market-oriented reform of healthcare. Many scholars in China gradually believe that ordered competition or management competition is the reasonable direction of market-oriented reform in the future, and increasing evidence also suggests positive effects of them [60–62]. For example, prospective payment reform in the medical insurance payment system leading by the government, such as diagnosis-related groups (DRGs), and the pricing reform of medical services items, would help to eliminate the financial motivation of suppliers to take “unwanted behavior” when facing competition pressure, avoiding the negative effect brought by the market-oriented reform [63]. In addition, more empirical evidence, especially for longitudinal studies based on patient individual-level data, should be provided in the future to give the implication for the government. Some market-oriented policies implemented in recent years also provide the opportunities to design “natural experiments” to infer causal effects, then provide more strongly supporting evidence for the solving of the debate about market-oriented reform in China.

## Efforts to inform efficient resources allocation

Health technology assessment (HTA) is a multidisciplinary activity that uses explicit methods to determine the value of a health technology (pharmaceutials in particular) at its different lifecycle points to inform healthcare decision making for promoting an equitable, efficient, and high-quality health system [64]. Since introduced to China in the 1980s, HTA has gradually played a role in China’s healthcare decision making in better allocate health technologies. The first HTA center founded at Shanghai Medical University in 1994, with supports from the Ministry of Health (MOH), technical assistances from the World Bank Loan Project and international HTA experts. From 1994, HTA in China experienced a continuous “nature” development in technology assessment, capacity building, and case-based policy translation, but had very little HTA institutional achievements tightly linked with policy making until two national HTA centers established under the National Health Commission (NHC) and the National Healthcare Security Administration (NHSA) in 2018 and 2019. Beyond two national HTA centers set up by the central government, there are dozens of HTA centers/units at the different levels of government affiliated research or administration centers, universities, some professional associations, public hospitals, foundations and consultative companies in different years. In addition, there are many potential HTA allied groups, such as evidence-based medicine, health system and policy research. HTA is of importance on the macro level that could help to inform policy- or decision-making and to formulate policy. With the policies and programs to address rising health care costs associated with pharaceuticals and health technologies, HTA in China has gradually shifted from its pure academic research to policy- or decision-oriented research, which plays an essential role in health project evaluation, resource allocation and reimbursement drug/services list adjustment. There are many challenges to be addressed in the development of health technology assessment. For example, there is a lack of explicit policy mechanism of implementing HTA to support healthcare decisions. As anticipated, the greater potential of HTA developing in China in its influence on healthcare decision making is yet to achieved till the NHSA initiated the price negotiations for the national drug reimbursement lists in 2017. There is a short supply of HTA capacity in technical staff conducting HTA research and making an appraisal of HTA evidence. Additionally, there is a lack of data infrastructure and accessibility for supporting a robust HTA system in China. Finally, national-level HTA institutionalization has yet to be established to develop standards for implementing HTA and coordinate the scope of HTA among decision-making concerning regulatory, pricing & reimbursement, and hospital and clinical settings.

## Building a reliable and sustainable pharmaceutical system in China

### Manufacturing capability

The pharmaceutical system is one key building block of health systems [65], especially in China, which has a huge population. Since the foundation of the PRC in 1949, China had faced the challenges of drug shortages for its health system for a long time. Back then, due to a historical lack of industrial basis, China could not even produce the most basic drugs. The startup development of the drug manufacturing capacity heavily relied on the Soviet Union's aid, which ended abruptly in 1960 due to the split between the Soviet Union and China. After that, the domestic pharmaceutical industry experienced a long-term stagnation in improving its manufacturing capacity independently until the Reform and Open-up of China in the 1980s. In the 1990s, China began to upgrade its manufacturing capabilities through multiple channels, including experimenting joint ventures with foreign pharmas, direct procurement of manufacturing equipment and techniques from Western countries, and encouraging private investment into drug manufacturing, etc. [66] With these efforts, the early twenty-first century has witnessed a dramatic improvement in the manufacturing capabilities of generic drugs in China to meet its major domestic demand.

### Innovative drugs

Compared with the improvement in generic drug supply, the research, development, and manufacturing of innovative drugs in China started even later. Since the 1990s, China had set catch-up strategies of “me-too”, “me-better”, and “fast-follow” in innovative drugs. With the support of the *National New Drug Innovation Project*, China founded more than 10 national comprehensive technology platforms, established eight firm technology innovation systems of drugs, arranged 10 crucial major disease plans (including malignant tumours, viral infectious diseases, cardiovascular diseases, autoimmune diseases, and diabetes, etc.). In addition, with strong policy support of the financial market (like IPO), China provided incentives for biopharmaceutical entrepreneurs and scientists from overseas to bring financial investment and frontier technologies to China to open their businesses [67, 68]. Through these works, China has stepped upwards from “me-too” to “me-better” and “fast-follow” in innovative drugs. For example, in the areas of immunotherapeutic drugs like PD-1/PD-L1, and cutting-edge gene and cell therapies, Chinese pharmaceutical companies are able to catch up with the international frontiers and promote their commercialisation in the global market. However, the innovation of first-in-class drugs remains a shortcoming of China.

### Essential medicine policy

While China vigorously improved its capability to supply generic drugs and began to progress in “me-too” and “me-better” drugs in the 2000s, its health system was widely criticised for its lack of unaffordable drugs. At the time, public hospitals used the 15% drug price-adding policy to increase hospital revenues, and doctors were motivated to prescribe high-priced and unnecessary drugs to patients. Consequently, patients and their families were overwhelmed by huge drug expenditures or even pushed or back into poverty because of illness, which gradually aroused wide concern among the public and the government. So, in August 2009, China initiated the essential medicine policy as one of the pillars of its comprehensive health reform [69]. The national health ministry established the *National Essential Medicine List*, composed of the most commonly used drugs in public hospitals and primary care institutions. According to the essential medicine policy, all the public medical institutions at different levels were required to procure and store essential medicines. Public hospitals and primary care should prescribe essential medicine to patients without any price-adding from the procurement price. Moreover, to ensure the effective implementation of the essential medicine policy, the government health departments at different levels would conduct annual assessments of the utilisation rate of essential medicine as one of the key performance indicators for public hospitals and primary care. With continuous enforcement and modifications, the essential medicine policy gradually took root in China’s health system, improving drug equity for patients to some extent.

### Drug reimbursement policy

Simultaneously, China initiated the *National Drug Reimbursement List* policy to support the implementation of the essential medicine policy and manage public medical insurance access to innovative drugs. More recently, China founded the National Healthcare Security Administration (NHSA) to consolidate responsibility and power for public health insurance funding and reimbursement (payment), including dynamically adjusting the list of drugs covered by medical insurance [70]. In terms of the entry of innovative drugs into the NDRL, NHSA has set the mechanism of drug price negotiation. Pharmaceutical companies are required to submit documents about pharmacology, clinical evidence, and pharmacoeconomics of targeted medicines, while NHSA will conduct independent technological and pharmacoeconomics reviews. Following these reviews, the NHSA would conduct face-to-face price negotiations with relevant pharmaceutical companies. A successful price negotiation often results in a 40–50% price reduction from the original price proposed by pharmaceutical companies. In addition to the price negotiation policy for innovative drugs, NHSA has also introduced the centralised drug procurement policy for generic drugs. This policy requires pharmaceutical companies to bid for the drug orders of the public health system. To take lead among competitors, successful bids always mean significant price reductions from existing market prices, sometimes even reaching 80%-90% of original prices. With the price negotiation and centralised procurement policy, NHSA has managed to realise its objectives to control and lower drug costs for China’s health system.

## Prospective sustainability challenge for China’s pharmaceutical system

Through consecutive policy efforts and reform over decades, China has established a relatively reliable pharmaceutical system to supply most generic drugs and some innovative drugs for its population. The number of registration and approval of innovative drugs and generic drugs changes from 2018 to 2022 in Table 1.However, the sustainability of its pharmaceutical system remains an issue. Notably, strong price control policies in recent years have been hindering the development of innovative drugs in the domestic market, in particular the “first-in-class” drugs, while increasing foreign drugmakers’ hesitancy to commercialise their most innovative drugs to China.

**Table 1:** The number of innovative drugs and generic drugs during 2018–2022

Year	Innovative drugs		Generic drugs	
	Clinic	Product	Clinic	Product/Market
2018	–	–	58	464 (P)
2019	–	–	107	373 (P)
2020	1096	20	41	722 (P)
2021	1522	45	52	933 (M)
2022	1615	18	–	–

The data from https://www.nmpa.gov.cn/directory/web/nmpa/index.html

## Conclusion

Chinese national health system holds the prominent place in the wider Global South and LMICs nations landscape. Huge size of national economy recently exceeded that of entire European Union [71, 72]. Chinese international trade with Association of Southeast Asian Nations (ASEAN) nations and wider South-East Asia is accelerating its growth after the recovery of trade routes. In terms of health sector this means that global demand and supply of medical goods, services and pharmaceuticals remains largely driven by Chinese domestic developments [73, 74]. Two additional factors underling these circumstances. First is the fact that Chinese pharmaceutical market has in recent years replaced the Japanese one after many decades as the leading Asian giant [75]. Second is the fact that the pool of middle-class citizens as the driver of overall real gross domestic product growth continues to expand in all of the BRICS nations. Incremental gains in purchasing power remain most massive in East Asian societies. This fact is in sharp contrast with contracting household purchasing power and percentage share of middle-class citizens across an array of wealthy OECD countries 37 [76]. Furthermore, Chinese domestic manufacturing and sales of decent quality medical technologies and services has grown exponentially. This was the case particularly in late 2010s and early 2020s [77]. In order to gain broader understanding of the underlying drivers of Chinese health sector development we had to look deeper into the past. Some temporary pitfalls and increasing in rural–urban inequalities in equity of access and affordability of medical care and pharmaceuticals did take place [78]. Yet despite these difficulties to generate balanced development strategy for the largest global market, we think that this is a clear path upwards. Its success story was documented above in terms of struggle against poverty. Further upcoming improvements expanding health insurance coverage are in strong demand for certain layers of the society. Domestic bottleneck weakness yet remains manufacturing, import and market penetration of cutting-edge pharmaceuticals. Yet some of these obstacles are likely to be overcome in foreseeable future with the adoption of responsible strategies by governmental agencies in health care arena.

## Data Availability

Not applicable.
